# Effects of Viable and Heat-Inactivated *Bifidobacterium longum* D42 on Proliferation and Apoptosis of HT-29 Human Colon Cancer Cells

**DOI:** 10.3390/foods13060958

**Published:** 2024-03-21

**Authors:** Yifan Zhang, Ting Cao, Yuqi Wang, Rui Yang, Yu Han, Shuang Li, Deyu Liu, Yingxue Yue, Yun Cao, Bailiang Li, Song Wang, Guicheng Huo

**Affiliations:** 1 Key Laboratory of Dairy Science, Ministry of Education, Northeast Agricultural University, Harbin 150030, China; 2 Food College, Northeast Agricultural University, Harbin 150030, China

**Keywords:** *Bifidobacterium longum*, heat-inactivated probiotic, colon cancer, HT-29 cell, proliferation, apoptosis

## Abstract

*Bifidobacterium longum* is a common probiotic; both viable and heat-inactivated *Bifidobacterium longum* have many probiotic effects, such as anticancer effects. But some mechanisms of anticancer effects are still unclear, especially for heat-inactivated probiotics. In this study, we analyzed the effects of viable and heat-inactivated *Bifidobacterium longum* D42 on human colon cancer cells (HT-29). Cell proliferation, membrane permeability and apoptosis were detected by using the CCK-8 method, LDH method and Annexin V-FITC/PI kits. The ROS level and mitochondrial membrane potential were examined using the fluorescent probes DCFH-DA and JC-1. Real-time fluorescence quantitative PCR (RT-qPCR) and Western blot were used to detect the expression of mitochondrial apoptosis pathway genes and proteins. The results showed that viable and heat-inactivated *Bifidobacterium longum* D42 at concentrations of 1 × 10^6^ CFU/mL significantly inhibited the proliferation of and increased the level of LDH release of HT-29 colon cancer cells. We found that they could increase the apoptosis rate of HT-29 cells. Moreover, they could also induce apoptosis by inducing cells to produce ROS and destroying the mitochondrial membrane potential of cells. Further studies found that they could increase the mRNA transcription and protein expression levels of the Caspase-3, Caspase-9 and Bax genes in cells, and reduce the mRNA transcription and protein expression levels of the Bcl-2 gene. In summary, our findings revealed that viable and heat-inactivated *Bifidobacterium longum* D42 have inhibitory effects on proliferation and promote the apoptosis of human colon cancer cells, and also have certain adjuvant drug therapeutic effects and have potential application value in the adjuvant treatment of colon cancer.

## 1. Introduction

Colon cancer is the third most common malignant tumor and the second most common cause of death worldwide [1]. In recent years, the incidence rate of colon cancer in China has been rising rapidly and this trend is predicted to continue in the next few years [2]. The occurrence and development of colon cancer is a complicated process influenced by multiple factors like genetic factors, environment, and lifestyle; however, most cases of colon cancer can be due to lifestyle and aging, while a few cases are attributed to underlying genetic diseases [3]. In addition, research over the past few decades has shown that the role of the intestinal microbiota is becoming more and more important in health and disease, and the occurrence of various diseases is related to biological dysbiosis in the gut [4]. Changes in dietary habits or ecological factors can influence changes in the intestinal microbiota, which can disrupt the gut microbiota and increase intestinal permeability, leading to colitis and potentially leading to the initiation and development of colon cancer [5]. Historically, surgery, radiation therapy, chemotherapy, and immunotherapy have been the main methods of treating colon cancer. Surgery is one of these therapeutic options that is appropriate for early patients with colon-limited lesions; however, 20% of patients with colon cancer have distant metastases at the time of diagnosis and are therefore not eligible for surgery [6]. The primary treatment option for colon cancer patients is chemotherapy. However, chemotherapy drugs such as 5-fluorouracil (5-FU) may often cause symptoms such as nausea, vomiting, intestinal damage, decreased immune function, and even lead to drug resistance, causing recurrence [7]. Therefore, some biological agents have been of great significance in regulating the composition of gut microbiota and the immune system response, as well as preventing and providing support during treatment.

Probiotics are defined as “viable microorganisms that are beneficial to the host when they reach a certain number.” [8]. Probiotics have some recognized beneficial effects on human health, including regulating immunity, reducing cholesterol, anticancer, anti-diarrhea, anti-diabetes and anti-hypertension effects, reducing blood lipids and improving lactose metabolism, etc. [9]. Currently, many studies have confirmed that probiotics may contribute to the host’s potential resistance to developing colon cancer. Probiotics can play an anticancer role by inhibiting cancer cell proliferation, inducing cancer cell apoptosis, enhancing the immune system, improving the intestinal barrier and resisting oxidation [10]. In addition, probiotics have a certain preventive effect on colon cancer. A randomized trial conducted on 398 male and female participants showed that the incidence of moderate and highly atypical tumors was lower after taking *Lactobacillus casei* [11]. However, some studies suggested that probiotics have certain side effects on the treatment of colon cancer, such as causing gastrointestinal disorders in colon cancer patients (bloating, ecological imbalance of gut microbiota and the spread of resistance genes between gut microbiota) [12]. In addition, some probiotics may have a negative regulatory effect on the immune system [13]. To overcome the potential adverse effects of using live probiotics, some potential alternative ingredients, such as prebiotics (“a substrate that is selectively utilized by host microorganisms conferring a health benefit” [14]) and postbiotics (“preparation of inanimate microorganisms and/or their components that confers a health benefit on the host” [15]), have been applied to balance the components of intestinal microbiota, create suitable intestinal environmental conditions and maintain dynamic balance in development. Heat-inactivated probiotics, as a type of postbiotics, have many advantages, such as higher safety, longer shelf life, low cost and better stability [16]. They can be used as potential anticancer drugs for adjuvant therapy in colon cancer patients.

Some studies have shown that heat-inactivated probiotics have an inhibitory effect on colon cancer. Heat-inactivated *Lactobacillus brevis* and *Lactobacillus paracasei* have been shown to clearly inhibit the growth of HT-29 cells and induce their apoptosis, and Lactobacillus brevis has a stronger inhibition and apoptosis-inducing ability than *Lactobacillus paracasei*, with no obvious toxicity to normal HEK-293 cells [17]. The inhibition rate of SW480 cells reached 28.49% with treatment with heat-inactivated Saccharomyces cerevisiae, with a higher inhibitory effect than the anticancer drug 5-FU [18]. In addition, heat-inactivated probiotics can exert anticancer effects by participating in immune regulation. Chung found that Caspase-1 activity and IL-1β maturity were reduced by the heat-inactivation of *Enterococcus faecalis*, thus inhibiting the activation of NLRP3 inflammatory bodies in macrophages; in addition, the strain could improve the severity of intestinal inflammation, thus protecting wild-type mice from the formation of colitis and colorectal cancer induced by sodium dextran sulfate (DSS) in vitro experiments [19].

*Bifidobacterium longum* is the most common species of *Bifidobacterium* found in the gut microbiota of infants and adults, playing an important role in human health. It has physiological functions such as inhibiting intestinal pathogenic bacteria activity, and also displays antioxidant activity and immunomodulatory activity [20]. A study showed that *Bifidobacterium longum* isolated from breast milk can relieve colon cancer by significantly increasing the concentration of IL-1β, an anti-inflammatory factor, and decreasing the concentration of IL-6, a pro-inflammatory factor in colon cancer model mice [21]. In addition, feeding rats with *Bifidobacterium longum* freeze-dried culture medium could significantly reduce the volume of colon tumors, reduce the incidence of colon cancer and inhibit the cell proliferation induced by carcinogens (azomethane), the activity of ornithine decarboxylase associated with colon adenoma and cancer, and the expression of ras-p21 cancer protein [22]. At present, there are some studies on the effect of live *Bifidobacterium longum* on relieving colon cancer, but there are still few studies on the effect of heat-inactivated *Bifidobacterium longum* on inhibiting colon cancer. Consequently, this study aims to investigate the cytotoxic effects of viable and heat-inactivated *Bifidobacterium longum* on HT-29 cells, providing a basis for the development of functional viable and heat-inactivated *Bifidobacterium* with preventive effects on colon cancer. Meanwhile, this study has completed the groundwork for in vivo research on the inhibitory effects of live and heat-inactivated *Bifidobacterium* on colon cancer.

## 2. Materials and Methods

### 2.1. Strain Culture, Cell Culture and Materials

*Bifidobacterium longum* D42 used in this study were preserved in Northeast Agricultural University and incubated into mMRS broth (MRS broth containing 2% *v*/*v* L-cysteine hydrochloride) at 37 °C for 48 h under oxygen-free conditions. The strain was passage cultured twice for 24 h, and, to obtain heat-inactivated *B. longum* D42, the strain was heated at 65 °C for 30 min. The spread plate method was used to determine the number of viable bacteria and to judge whether thermal inactivation was complete. Then, viable and heat-inactivated *B. longum* D42 were centrifuged (8000× *g*, 10 min) and washed with PBS buffer twice. After washing steps, the strains were resuspended with cell culture medium (antibiotic-free) to achieve a final concentration of 1 × 10^6^ CFU/ mL, and viable and heat-inactivated *B. longum* D42 (D42 and HID42) were directly used in subsequent experiments.

HT-29 cells were purchased from Qingqi Biological Co., Ltd. (Shanghai, China). HT-29 cells were cultured in high-glucose Dulbecco’s modified Eagle medium (DMEM) supplemented with 10% fetal bovine serum (FBS) and 1% antibiotics (100 U/mL penicillin and 0.1 mg/mL streptomycin), then cultured at 37 °C in a humid atmosphere containing 5% CO_2_ [23]. Cultural media, the CCK-8 Assay Kit and the Annexin V/PI Staining Kit were procured from MeilunBio (Dalian, China), the LDH Assary Kit and ROS Assay Kit were sourced from Beyotime (Shanghai, China). Additionally, fluorescence quantitative PCR experiment-related kit was purchased from APExBIO (Houston, TX, USA).

### 2.2. Proliferation Analysis of HT-29 Cells

Inhibition rate was assessed using the CCK-8 Assay Kit. HT-29 cells were inoculated into a 96-well plate at 1 × 10^5^ cells/mL and exposed to the viable and heat-inactivated *B. longum* D42 group for 24 h, respectively, and 5-FU (100 μg/mL) was used as a positive control. In addition, the two groups (5-FU and viable *B. longum* D42 group, named 5-FU+D42; 5-FU and heat-inactivated *B. longum* D42 group, named 5-FU+HID42) were designed, respectively. After the reaction, the cells containing 110 μL of the diluted CCK-8 solution (100 μL of cell culture medium and 10 μL of CCK-8 solution) were cultured in a cell incubator for 1~2 h until the liquid turned orange and the absorbance was measured at a wavelength of 450 nm.

Inhibition rate (%) = (Ac − As)/(Ac − Ab) ×100%

Ac: The absorption of the control cells.

As: The absorption of the different treated cells.

Ab: The absorption of the blank hole with cultural medium.

### 2.3. Measurement of Lactate Dehydrogenase Release in HT-29 Cells

HT-29 cells were inoculated into a 96-well plate at the concentration of 1 × 10^5^ cells/mL, then five test groups (D42 group; HID42 group; 5-FU group; 5-FU+D42 group; and 5-FU+HID42 group) were added to the cell culture for 24 h, respectively. After reaching the predetermined time, the supernatant of each well was taken, added to LDH detection working solution, mixed evenly, incubated in the dark at room temperature for 30 min, and then the absorbance was measured at 490 nm. The amount of LDH released by cells in the blank group was set to 100% and compared with that released by other treatment groups.

### 2.4. Morphological Analysis of Apoptosis in HT-29 Cells

HT-29 cells were inoculated into a 6-well plate at the concentration of 1 × 10^5^ cells/well, then five test groups (D42 group; HID42 group; 5-FU group; 5-FU+D42 group; and 5-FU+HID42 group) were applied to the cell culture for 24 h, respectively. After draining the culture solution from the 6-well plates, 0.5 mL of fixative was applied and left for 10 min. A total of 1 mL of PBS was used twice for three minutes of washing each time after removing the fixative. To each well was added 0.5 mL Hoechst 33258 dyeing solution and the cells were stained for 5 min. Then, the liquid was drained and the cells were cleaned twice with 1 mL of PBS for 3 min at a time, and the liquid was discharged. Finally, an appropriate amount of solution for anti-fluorescence quenching was dripped into the well plate, and the cells were observed with a fluorescence microscope.

### 2.5. Apoptosis Analysis of HT-29 Cells

HT-29 cells were treated in the same way as the morphology analysis. After the end of culture, the cells were digested, centrifuged at 4 °C for 5 min at 1000 rpm and the supernatant was discarded, cleaned twice with PBS buffer and the precipitate was left. The cells were blended by adding 200 µL diluted Binding buffer working solution and 5 µL Annexin V-FITC, and cultured in the dark at room temperature for 15 min. Then, 5 µL propidium iodide staining solution and 300 µL diluted Binding buffer working solution were added, and mixed gently. The cells were detected by flow cytometry.

### 2.6. Determination of Reactive Oxygen Species Level in HT-29 Cells

HT-29 cells were inoculated into 6-well plates at 1 × 10^5^ cells per well and were treated with viable *B. longum* D42 and heat-inactivated *B. longum* D42 for 24 h, respectively. The cell culture solution was removed from plates. To each well was added an appropriate amount of DCFH-DA, and then they were placed in a cell incubator at 37 °C for 20 min. The cells were cleaned three times with serum-free medium and observed under a fluorescence microscope.

After centrifugation, cells in each well were centrifuged at 1000 rpm for 5 min, collected in a centrifuge tube, washed with PBS buffer twice and with serum-free cell culture medium the third time. DCFH-DA was diluted to prepare a solution and suspend the washed cells, which were then cultured in a cell incubator for 20 min. After incubation, the cells were centrifuged and cleaned with serum-free cell culture medium three times, resuspended in 1 mL PBS buffer, and 100 μL of resuspended cells was added to a 96-well plate. The fluorescence spectrophotometer with excitation wavelength of 488 nm and emission wavelength of 525 nm was used to test the plate.

### 2.7. Determination of Changes in Mitochondrial Membrane Potential in HT-29 Cells

HT-29 cells were inoculated into 6-well plates at 1 × 10^5^ cells per well and were treated with viable *B. longum* D42 and heat-inactivated *B. longum* D42 for 24 h, respectively. All wells were cleaned twice with 1 mL PBS, and to each was added 1 mL of cell culture medium and JC-1 dyeing working solution, then they were cultured for 20 min in a 37 °C cell culture incubator. The supernatant was removed after incubating, and cleaned with JC-1 staining buffer (1×) twice. A total of 2 mL cell culture medium was added to the cells before observation under a fluorescent microscope.

The cells were taken out and mixed in 0.5 mL cell culture media in order to be detected using a fluorescence spectrophotometer. After that, to the cells was added 0.5 mL of JC-1 staining solution, then they were resuspended several times and incubated for 20 min at 37 °C in a cell incubator. After centrifuging the cells (1000 rpm, 4 °C) for 3 min, the cells were twice washed with JC-1 staining buffer (1×). At last, the cells were reconstituted in a suitable quantity of JC-1 staining solution (1×), and a fluorescence spectrophotometer was used to detect them.

### 2.8. Analysis of Expression Level of Apoptosis-Related Genes in HT-29 Cells

HT-29 cells were plated in culture plates of Φ6 at 1 × 10^5^ cells each well and were treated with five different conditions (D42 group; HID42 group; 5-FU group; 5-FU+D42 group; and 5-FU+HID42 group) for 24 h. Total RNA of cells was extracted according to the instructions of the RNA extraction kit, and the extracted RNA was synthesized into cDNA according to the reverse transcription kit instructions. The reaction system (total volume of 20 μL) was mixed on ice, and then the reverse transcription reaction was carried out on a PCR instrument at 25 °C for 10 min, 42 °C for 50 min and 95 °C for 15 min to obtain cDNA. The instructions of the Talent qPCR PreMix (SYBR Green) kit were then followed. The total volume of the reaction system was 20μL, and it was pre-denatured at 95 °C for 2 min, denatured at 95 °C for 15 s, and extended at 60 °C for 30 s. After 40 cycles of denaturation and extension, the melting curve was analyzed. The primers needed for the experiment are shown in Table 1. 

Calculation method: GAPDH was used as internal reference gene, and the comparative expression of the target gene was calculated by 2^−ΔΔ^ cycle threshold relative quantitative method according to ΔCt= target gene Ct- reference gene Ct, ΔΔCt = ΔCt test group −ΔCt blank group in blank group and treatment group [24].

### 2.9. Analysis of Expression Level of Apoptosis-Related Proteins in HT-29 Cells

HT-29 cells were plated in cell culture plate of Φ6 at 1 × 10^5^ cells/well and were treated with five different conditions (D42 group; HID42 group; 5-FU group; 5-FU+D42 group; and 5-FU+HID42 group) for 24 h. The cells in each group were collected, then the proteins were extracted by using the protein extraction kit, and quantified by using the BCA protein concentration determination kit.

SDS-PAGE electrophoresis was carried out on the extracted protein samples, and the initial voltage was 60 V. When the protein samples entered the separation gel, the voltage was adjusted to 90 V. After that, semi-dry film transfer was carried out, and the film transfer condition was 25 V for 30 min. A 5% skimmed milk powder was used to seal the membrane for one hour at room temperature. The primary antibody was diluted to the required concentration according to the instructions, and incubated at 4 °C overnight. The HRP-labeled secondary antibody was diluted to 1:1000 and incubated at 37 °C for 1 h. Color development and image analysis: The correct amount of ECL luminescent liquid was taken and added, in the darkroom, to the front of the membrane. Light was avoided for 5 min. The film was then put into an imaging system for scanning. The results were analyzed by using the software Gel-Pro Analyzer 4.0.

### 2.10. Statistical Analysis

Every experiment was conducted three times independently, and the results were expressed as means and standard deviations. SPSS 25.0 software (SPSS Inc., Chicago, IL, USA) was used for one-way ANOVA and Duncan’s multiple comparative analysis. In this study, *p* < 0.05 indicated that the difference was statistically significant, and *p* > 0.05 indicated that the difference was not significant.

## 3. Results and Discussion

### 3.1. Effect of Viable and Heat-Inactivated B. longum D42 on the HT-29 Cells

**Figure 1:**
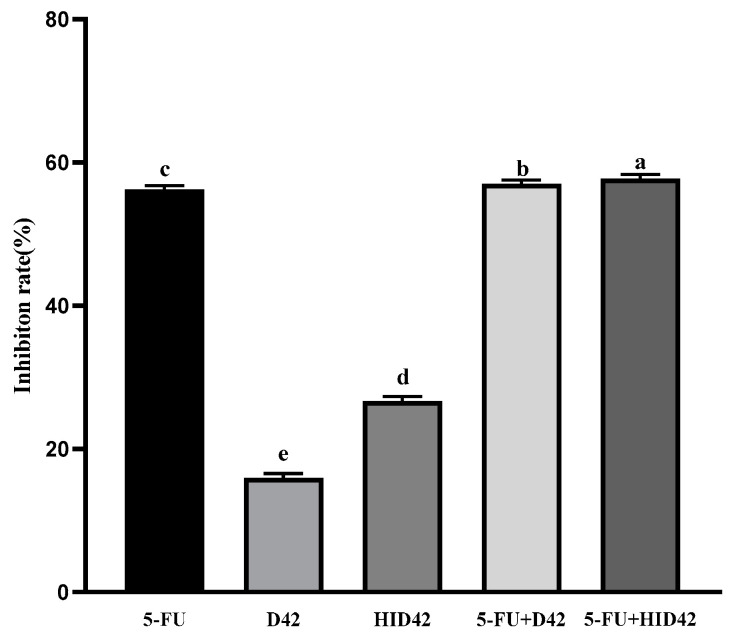
Inhibition of HT-29 cell proliferation after co-incubation with different subjects. Different lowercase letters (a–e) above the columns indicate significant data differences between different groups (*p* < 0.05).

The purpose of this study was to research the effect of viable *B. longum* D42 and heat-inactivated *B. longum* D42 on HT-29 colon cancer cells. Our results showed that each test group showed different degrees of inhibition of the proliferation of HT-29 cells (Figure 1). The HT-29 cells treated with D42 and HID42 all showed a clear inhibitory effect, and the inhibitory rates were 15.93% and 26.69%, respectively. The inhibition rate of the cells treated with HID42 was significantly higher than those treated with D42 (*p* < 0.05). In addition, compared with only adding D42 and HID42 and 5-FU, the inhibition effects of the 5-FU+D42 group and the 5-FU+HID42 group were more significant and the inhibitory rates were 56.97% and 57.77% (*p* < 0.05).

In recent years, probiotics have been widely studied as an effective biological agent to alleviate cancer, and the potential of heat-inactivated probiotics to alleviate cancer has gradually attracted significant attention. Many studies have shown that some viable probiotics and heat-inactivated probiotics can inhibit the proliferation of cancer cells. A study screened the HT-29 colon cancer cells by using 140 live strains of *Lactobacillus* obtained from baby feces and traditional fermented foods in Western China, and found that 11 of the viable strains displayed an anti-proliferation effect [25]. Besides viable probiotics, heat-inactivated probiotics also have anti-colon cancer functions. Both the viable and heat-inactivated *Escherichia coli Nissle* 1917 could produce toxic effects on HT-29 cells, and the toxic effects were enhanced with the increase in concentration and incubation time [26]. This study focused on the inhibitory effects of viable and heat-inactivated *Bifidobacterium longum* D42 on colon cancer cells, and described their characteristics as potential anticancer drugs, thus further emphasizing their potential for relieving cancer.

### 3.2. Effect of Viable and Heat-Inactivated B. longum D42 on the Membrane Permeability

**Figure 2:**
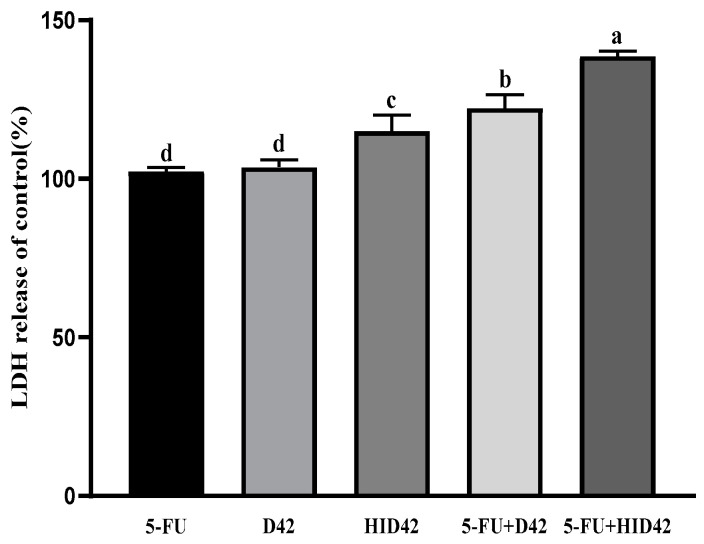
Variation in LDH release rate in HT-29 cells after different treatments. Different lowercase letters (a, b, c, and d) above the columns indicate significant data differences between different groups (*p* < 0.05).

Our results showed that each test group had different effects on LDH release from HT-29 cells (Figure 2). Compared with the control group, the release of LDH in the different treatment groups increased by different degrees. Among them, the release of LDH in the 5-FU treatment group was the lowest, which was 102%. The release of LDH in the D42 group and the HID42 group was higher than that in the positive control group, which was 104% and 115%, respectively. In addition, the release of LDH in the 5-FU+D42 and 5-FU+HID42 groups was significantly higher than that in the D42 group and the HID42 group, which was 122% and 139% (*p* < 0.05). Moreover, the effect of 5-FU+HID42 on LDH release is better than that of 5-FU+D42 (*p* < 0.05). The results showed that both viable and heat-inactivated *B. longum* D42 could destroy the cell structure of colon cancer and release LDH, which is consistent with the hypothesis of the preliminary study. Heat-inactivated *B. longum* D42 had a stronger effect on promoting LDH release, and both the viable and heat-inactivated *B. longum* D42 had the ability to increase the release of LDH by 5-FU.

In the case of apoptosis or necrosis, all kinds of cellular mechanisms are destroyed and the structure and functional integrity of cell membranes are affected. Therefore, some enzymes in the cytoplasm, such as lactate dehydrogenase (LDH), are released into the culture medium when cells are destroyed; the amount of LDH released can reflect the amount of cells with damaged cell membranes, and then reflect the amount of cells undergoing apoptosis or necrosis, so the amount of released LDH can be regarded as an indicator to quantify apoptotic or necrotic cells [27]. This experiment confirmed that both viable and heat-inactivated *B. longum* D42 could induce HT-29 cells to release LDH, destroy cell membranes and induce apoptosis. The same phenomenon was also discovered in the human colon cancer cell line SW480 after treatment with propofol; compared with the blank control group, the LDH activity of the cell line SW480 in the propofol treatment group was significantly increased, and cell proliferation was inhibited [28].

### 3.3. Effect of Viable and Heat-Inactivated B. longum D42 on Apoptosis of HT-29 Cells

Based on the above research results and ongoing research, we speculated that viable and heat-inactivated *B. longum* D42 could induce apoptosis of colon cancer cells. Therefore, we confirmed this conjecture by detecting apoptosis, the apoptosis rate, ROS level and changes in mitochondrial membrane potential.

**Figure 3:**
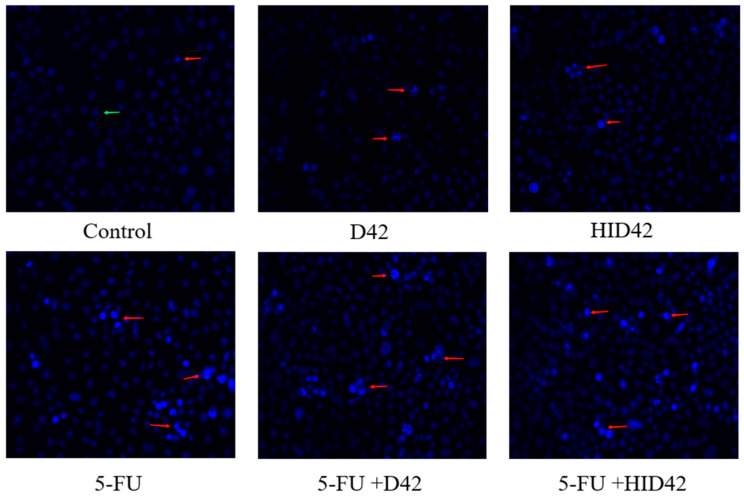
Hoechst 33258 staining was used to observe the morphology of HT-29 cells treated with different subjects. The red arrow indicates apoptotic cells and the green arrow indicates intact cells. Photographs were captured by using a fluorescence microscope (20×).

The morphological characteristics of the HT-29 cells were observed under a fluorescence microscope with Hoechst 33258 staining (Figure 3). After dyeing with Hoechst 33258 dye, the normal nuclei were usually light blue, and the extranuclear background was uniformly black, while the apoptotic nuclei were bright blue, and were usually densely stained in multiples or blocks. The HT-29 cells were small in size and uniform in shape, and the fluorescent body was bright after staining, so they were easy to identify by this method. Compared with normal control cells, the apoptotic bodies of the cells treated with D42 and HID42 all exhibited strong blue fluorescence. The cells treated with 5-FU+D42 and 5-FU+HID42 were stained more densely, and the fluorescence brightness of the cells was denser than that of the cells treated with 5-Fu. The results showed that viable and heat-inactivated *B. longum* D42 could not only promote the apoptosis of colon cancer cells, but also enable drugs to induce apoptosis.

**Figure 4:**
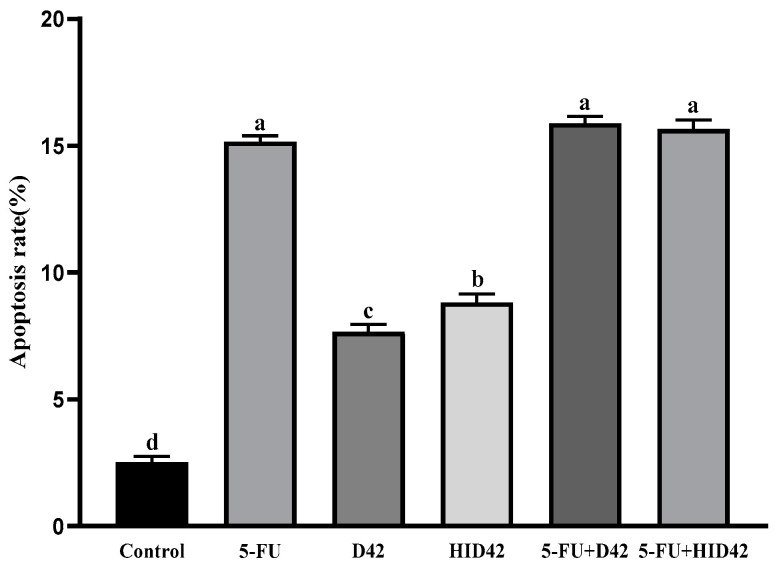
The apoptosis of HT-29 cells in each treatment group. Different lowercase letters (a–d) above the columns indicate significant data differences between different groups (*p* < 0.05).

The HT-29 cells were treated for 24 h with different conditions, and the apoptosis of the HT-29 cells was detected by flow cytometry (Figure 4). The results indicated that the apoptosis rates of the 5-FU group, the D42 group and the HID42 group were 14.9%, 8% and 9.2%, respectively, when they were co-cultured with HT-29 cells for 24 h, and there was a significant difference between the apoptosis rates of the D42 group and the HID42 group. The apoptosis rates of the D42 group and the HID42 groups were lower than that of the 5-FU group. The apoptosis rates of the 5-FU+D42 group and the 5-FU+HID42 group were significantly higher than that of the D42 group and the HID42 group, which were 15.8% and 15.3% (*p* < 0.05), but there was no significant difference compared with that of the 5-FU group (*p* > 0.05). The apoptosis rate of the different treatment groups varied, which is consistent with the results observed with Hoechst 33258 staining.

Apoptosis is regulated by cytokines and genes. Abnormal apoptosis is an important pathology of many tumors and an important therapeutic target of many drugs, including probiotics [29]. Inhibiting the proliferation of cancer cells and promoting the apoptosis of cancer cells is a means to treat cancer; therefore, the determination of apoptosis can help to judge the effect of experimental substances on cancer cells. Annexin V can selectively bind phosphatidylserine (PS). Most PS is found on the inside of cell membranes. At the point of early apoptosis, PS is turned out onto the cell surface, which is the outer side of cell membrane. Annexin V can exhibit green fluorescence when combined with PS that has been turned out onto the cell surface. Red fluorescence can be observed in necrotic cells or cells that lose their cell membrane integrity during the latter stages of apoptosis when stained with PI. Necrotic cells exhibit green fluorescence due to the loss of cell membrane integrity, which allows Annexin V-FITC to enter the cytoplasm and combine with PS inside the membrane [30]. Our study showed that both viable and heat-inactivated *B. longum* D42 had the ability to induce the apoptosis of HT-29 cells.

**Figure 5:**
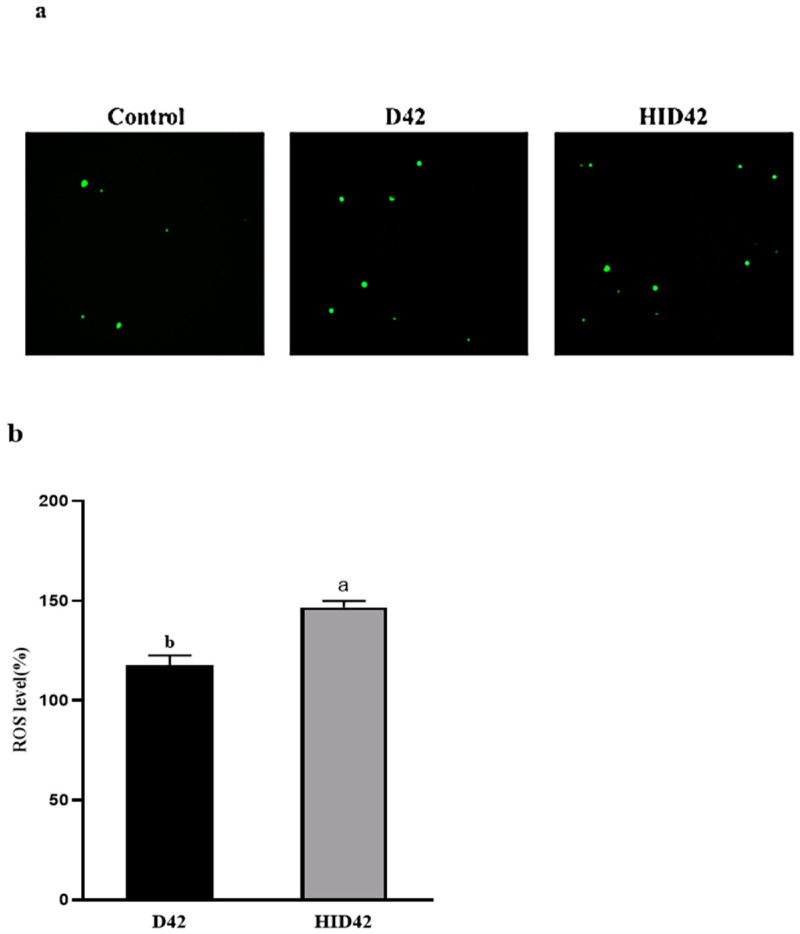
(**a**) ROS staining of HT-29 cells in each treatment group. Photographs were captured by a fluorescence microscope (10×); (**b**) change in ROS level in HT-29 cells after treatment with different conditions. Different lowercase letters (a and b) above the columns indicate significant data differences between different groups (*p* < 0.05).

Despite lacking fluorescence, DCFH-DA is able to pass through cell membranes with ease. It can be digested by esterase within the cell once it enters to produce DCFH. It is simple to insert the probe into the cell since DCFH is unable to pass through the cell membrane. The fluorescence of DCF can be used to determine the amount of reactive oxygen species present in cells, as these species can oxidize non-fluorescent DCFH to produce DCF with green fluorescence. The ROS level was measured quantitatively by using the fluorescent probe DCFH-DA (Figure 5). A fluorescence microscope with magnification of 10× was used to observe the cells. Compared with the normal control cells, the green fluorescent spots in the cells increased after treatment with D42 and HID42. And the ROS levels of the cells in the D42 group and the HID42 group were higher than those in the untreated group, which were 117.7% and 146.7%, respectively. Compared with the cells treated with D42, the ROS production level in the cells treated with HID42 was significantly improved (*p* < 0.05).

Reactive oxygen species (ROS) are one-electron reduction products of a class of oxygen, such as the superoxide anion (O_2_^−^), hydrogen peroxide (H_2_O_2_) and hydroxyl radical (·OH) [31]. A higher level of ROS can induce oxidative stress in tissues or cells, leading to cell damage, which can eventually activate both internal and external apoptosis pathways mediated by mitochondria and death receptors, aggravate mitochondrial damage and promote cell apoptosis [32]. Therefore, the detection of ROS level can effectively evaluate drug-induced cell damage and judge the effect of drugs on cell apoptosis. The HT-29 cells produced a lot of reactive oxygen species (ROS) after being treated with *Lactobacillus paracasei* M5L; at the same time, the activities of superoxide dismutase (SOD) and catalase (CAT) clearly decreased, which indicated that *Lactobacillus paracasei* M5L could induce the apoptosis of HT-29 cells through ROS production [33]. Our study found that viable and heat-inactivated *B. longum* D42 could induce ROS production in HT-29 cells, which is reasonable.

**Figure 6:**
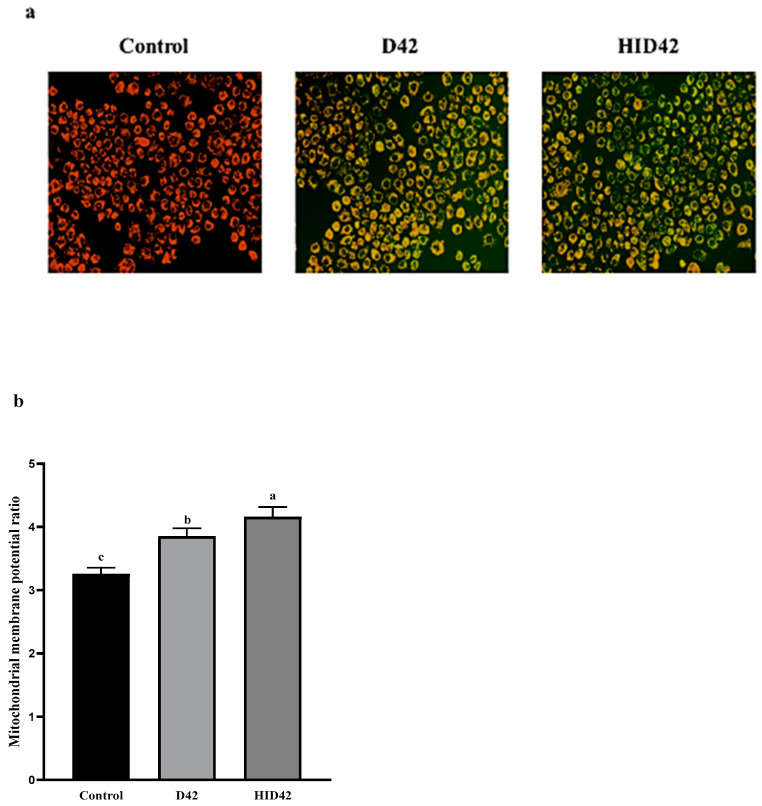
(**a**) Differences in mitochondrial membrane potential of HT-29 cells. Photographs were captured by using a fluorescence microscope (20×); (**b**) mitochondrial membrane potential ratio (green fluorescence/red fluorescence). Different lowercase letters (a–c) above the columns indicate significant data differences between different groups (*p* < 0.05).

We measured the changes in the mitochondrial membrane potential in HT-29 cells. The treated HT-29 cells were observed using a fluorescence microscope under 20× magnification and JC-1 staining (Figure 6). Green fluorescence was observed in the early apoptotic cells following JC-1 labeling, and at this time, the mitochondrial membrane potential was also in a reduced state. When the cells were normal, they showed red fluorescence after staining, and the mitochondrial membrane potential was normal at this point. Compared with the untreated cells, the cells treated with D42 and HID42 exhibited a further reduced mitochondrial membrane potential and produced obvious green fluorescence. The ratio of mitochondrial depolarization was determined by dividing the wavelength of green fluorescence by the wavelength of red fluorescence. This ratio was then utilized to indicate the change in the mitochondrial membrane potential. Compared with the D42 group, the change in the mitochondrial membrane potential in the HID42 group was more significant (*p* < 0.05). This result proved that the treatment with viable and heat-inactivated *B. longum* D42 could destroy the membrane potential of cancer cells and increase anticancer activity.

Apoptosis is a complex process of cell death, which is regulated by many factors, and reactive oxygen species (ROS) are one of them. Mitochondria is the main organelles responsible for energy metabolism, and a healthy mitochondrial membrane potential is essential for preserving the mitochondria’s ability to metabolize energy. Mitochondria will produce a large number of ROS when they are productive, and the over-expression of ROS will increase the permeability of mitochondria, change the mitochondrial membrane potential, damage mitochondrial DNA and cause oxidative damage to mitochondria [34]. The change in the mitochondrial membrane potential can affect the growth and apoptosis of cells. A sign of early apoptosis is the decrease in mitochondrial membrane potential; the changes in the mitochondrial membrane potential can reflect the degree of apoptosis. JC-1 is a probe commonly used in clinical research to detect the mitochondrial membrane potential. The polymer formed by JC-1 aggregation in the matrix of mitochondria will produce red fluorescence when the membrane potential of mitochondria is high. Since the potential of the mitochondrial membrane is low, JC-1 can produce green fluorescence as a monomer. The change in the fluorescence of JC-1 from red to green can be utilized as an early indicator of apoptosis and is a clear indicator of a drop in the cell membrane potential. A study has shown that treating HT-29 cells with *Lactobacillus acidophilus* CICC 6074 for 48 h led to a decrease in the mitochondrial membrane potential and induced apoptosis [35]. The results showed that viable and heat-inactivated *B. longum* D42 could effectively reduce the mitochondrial membrane potential of HT-29 cells, which was similar to previous studies. Thus, viable and heat-inactivated *B. longum* D42 could induce apoptosis of colon cancer cells HT-29 by damaging the mitochondria.

### 3.4. Further Study on the Apoptotic Mechanism of Viable and Heat-Inactivated B. longum D42 towards HT-29 Cells

The above results showed that viable and heat-inactivated *B. longum* D42 could induce apoptosis by increasing the level of ROS in cells and reducing the mitochondrial membrane potential, and both of them could mediate the mitochondrial apoptosis pathway. RT-qPCR is one of the most efficient, accurate and convenient technical means to analyze the transcription level expression of target genes [36]. RT-qPCR technology can be used to quantitatively analyze a specific nucleic acid sequence in the sample to be tested, and the expression of the studied gene can be analyzed according to the results, so as to explore the mechanism of action from the gene level. Western blot is an effective method to detect and visualize the expression of protein in tissues and cells. Western blot can be used to analyze the expression of the protein in the sample to be tested, and at the same time, it can indirectly reflect the gene expression of the protein, which can also help to analyze the mechanism involved in the gene level. Therefore, RT-qPCR was used to detect the expression of genes related to the mitochondrial apoptosis pathway and Western blot was used to detect the expression of apoptosis-related proteins as evaluation indicators to explore the mechanism behind this process from a genetic perspective.

**Figure 7:**
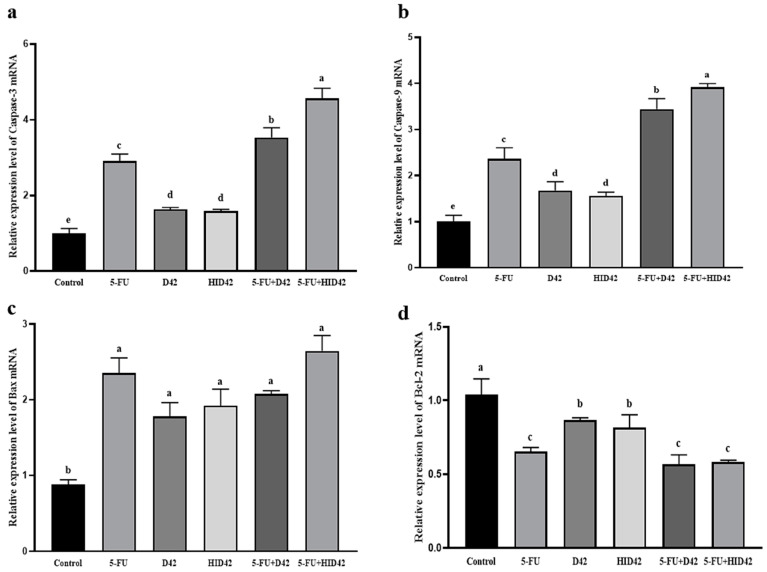
(**a**) The expression of apoptosis-related protein Caspase-3; (**b**) the expression of apoptosis-related protein Caspase-9; (**c**) the expression of apoptosis-related protein Bax; (**d**) the expression of apoptosis-related protein Bcl-2. Different lowercase letters (a, b, c, d and e) above the columns indicate significant data differences between different groups (*p* < 0.05) in each figure.

The different treatment groups had different degrees of regulation of mitochondrial apoptosis gene expression (Figure 7). Compared with the untreated group, the relative expression of Caspase-3, Caspase-9 and Bax mRNA in the other samples was increased significantly (*p* < 0.05) and the relative expression of Bcl-2 mRNA was decreased significantly (*p* < 0.05). Compared with the D42 group, there was no significant difference in the relative expression of Caspase-3, Caspase-9, Bax and Bcl-2 mRNA in the HID42 group (*p* > 0.05). Compared with the 5-FU group, the relative expression of Caspase-3 and Caspase-9 mRNA in the 5-FU+D42 group and the 5-FU+HID42 group was significantly increased (*p* < 0.05), but the relative expression of Bax and Bcl-2 mRNA showed no significant difference (*p* > 0.05).

**Figure 8:**
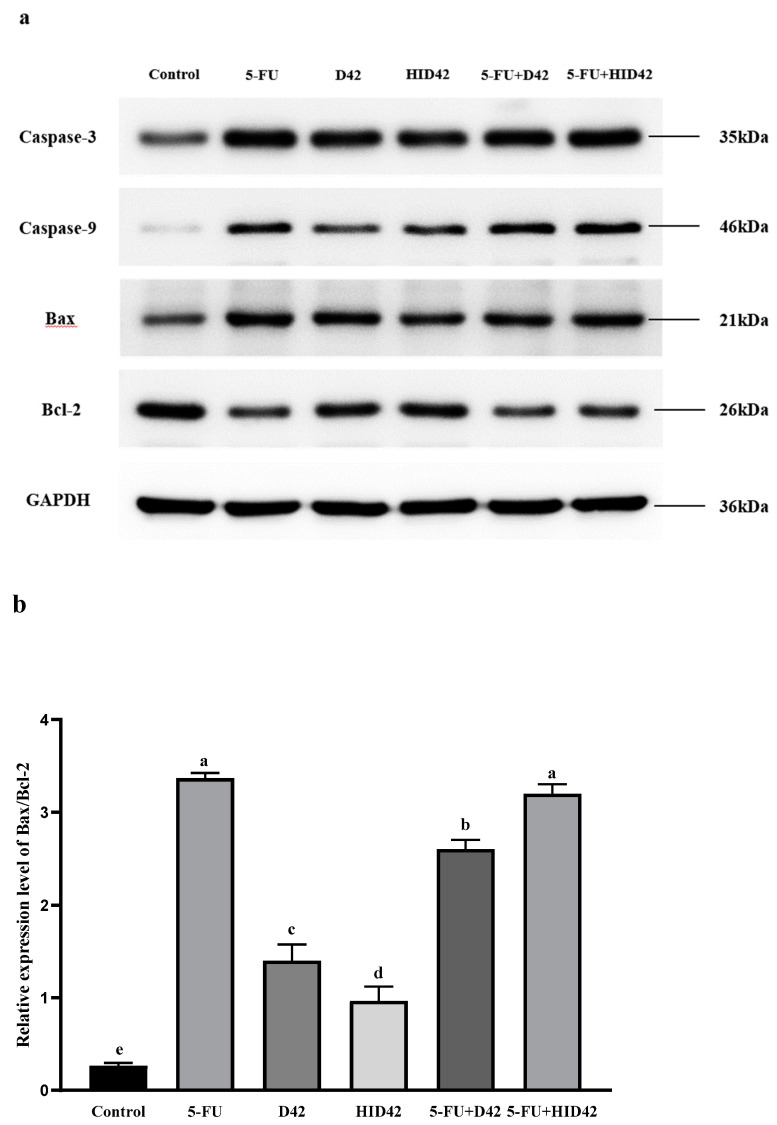
(**a**) The expression of apoptosis-related proteins in HT-29 cells was detected by Western blot; (**b**) the ratio of Bax/Bcl-2 in different treatment groups. Different lowercase letters (a–e) above the columns indicate significant data differences between different groups (*p* < 0.05).

The expression of apoptosis-related proteins detected by Western blot is shown in Figure 8. After the HT-29 cells were treated with 5-FU, the expression levels of Caspase-3, Caspase-9 and Bax protein increased significantly, while the expression level of Bcl-2 protein decreased significantly. Compared with the normal control group, the protein levels of Caspase-3 and Caspase-9 in the cells of the D42 group, HID42 group, 5-FU+D42 group and 5-FU+HID42 group increased. Compared with the normal control group, the ratio of Bax/Bcl-2 was significantly increased in the other treatment groups (*p* < 0.05). The ratio of Bax/Bcl-2 in the 5-FU+HID42 group was the highest, and there was no significant difference between it and the 5-FU group (*p* > 0.05), which was basically consistent with the findings of the RT-qRCR. The above results showed that the viable and heat-inactivated *B. longum* D42 could participate in the mitochondrial apoptosis pathway by regulating the expression of the Caspase-3, Caspase-9, Bax and Bcl-2 genes and proteins in the HT-29 cells, thus promoting the apoptosis of the HT-29 cells. In addition, viable and heat-inactivated *B. longum* D42 could help the chemotherapy drug 5-FU in the treatment of colon cancer.

At present, there are, primarily, two mechanisms involved in apoptosis: the endogenous (mitochondria) system and the exogenous (death receptor) pathway. A variety of stimuli, including oxidative stress and DNA damage, can activate the pro-apoptosis members of the Bcl-2 family on the mitochondrial membrane, leading to an increase in the permeability of the mitochondrial outer membrane and a decrease in the mitochondrial transmembrane potential, promoting the release of many pro-apoptosis proteins such as Cytochrome c (Cyt c), and activating caspase-9 and caspase-3, and the final process of cell apoptosis is the mitochondrial apoptosis pathway [37]. Bax is a pro-apoptosis factor of the Bcl-2 family, which can induce the release of Cyto-C, activate procapase-9 and start the cascade reaction of apoptosis. The inhibition of apoptosis factor Bcl-2 can inhibit the release of Cyto-C induced by Bax, thus preventing the apoptosis of cells [38]. Probiotics can enhance the role of pro-apoptotic proteins (such as Bax) involved in the cell cycle, down-regulate anti-apoptotic proteins (such as Bcl-2), and induce tumor cell apoptosis, thus preventing the occurrence and development of colon cancer. A study showed that the ratio of Bax/Bcl-2 in HT-29 cells was 1.19 after co-culture of *Lactobacillus* BCRC17010 and HT-29 cells, which was statistically significant compared with other control groups (*p* < 0.05), so it was inferred that *Lactobacillus* BCRC17010 could promote the apoptosis of HT-29 cells [39]. Our study supported the previous observation that viable and heat-inactivated *B. longum* D42 could induce the apoptosis of HT-29 cells by regulating the apoptosis factors of the mitochondrial pathway, and had a certain potential to assist the action of the anticancer drug 5-FU.

Although viable and heat-inactivated *B. longum* D42 showed different effects in terms of different indexes, both of them can evidently kill HT-29 colon cancer cells, which suggests that the bacterial components may play an anticancer role. At present, some studies have proved that some bacterial components of probiotics have anticancer effects. Heat-treated cells, crude cell walls and intracellular extracts from *Lactobacillus casei* 01 can significantly reduce the activity of HT-29 (*p* < 0.05) and play an anti-proliferation role [40]. A study showed that cell wall protein fractions from *Lactobacillus paracasei* can inhibit the proliferation of the human colon cancer cell line Caco-2 and promote apoptosis [41]. Heat-inactivated probiotics can promote the production of Th1-related cytokines and reduce Th2-related cytokines to participate in regulating the immune system [42]. The difference in the immunomodulatory activity may be related to the difference in the microbial cell wall components, such as peptidoglycan and lipoteichoic acid [16]. In this study, we found that viable and heat-inactivated *B. longum* D42 can promote the apoptosis of cancer cells by regulating the expression of some genes in mitochondrial apoptosis pathway, but the specific anticancer components and their effects on other immune-related pathways need to be studied further. In addition, in vivo experiments can be considered to further explore the mechanism of action.

## 4. Conclusions

In a word, our study found an inhibitory effect of viable and heat-inactivated *B. longum* D42 on HT-29 colon cancer cells. The results showed that viable and heat-inactivated *B. longum* D42 could inhibit the proliferation of colon cancer cells, induce the release of LDH, promote the apoptosis of cancer cells, increase the level of ROS in cancer cells, change the mitochondrial membrane potential of cancer cells and regulate the expression of genes and proteins related to the mitochondrial apoptosis pathway. Based on the above findings, we have confirmed that viable and heat-inactivated *B. longum* D42 have a clear effect of inhibiting proliferation and promoting the apoptosis of HT-29 colon cancer cells, and at the same time, they exhibit a certain adjuvant effect on anticancer drugs (5-fluorouracil). This discovery can not only provide a reference for the development and utilization of probiotics as an effective biological agent for inhibiting colon cancer, but can also provide a reference for research on the anti-colon cancer function of heat-inactivated probiotics. At the same time, it provides a theoretical basis for probiotics and heat-inactivated probiotics to be used as biological agents to assist clinical anticancer drug treatments.

## Figures and Tables

**Table 1:** Primers of RT-qPCR.

Gene	Forward Primer Sequence (5′-3′)	Reverse Primer Sequence (5′-3′)
Caspase-3	TGCCTGTAACTTGAGAGTAGATGG	CTTCACTTTCTTACTTGGCGATGG
Caspase-9	TGCTGCGTGGTGGTCATTCTC	CCGACACAGGGCATCCATCTG
Bax	CCCGAGAGGTCTTTTTCCGAG	CCAGCCCATGATGGTTCTGAT
Bcl-2	GGTGGGGTCATGTGTGTGG	CGGTTCAGGTACTCAGTCATCC
GAPDH	AAGCTCATTTCCTGGTATGACAACG	TCTTCCTCTTGTGCTCTTGCTGG

## Data Availability

The data presented in this study are available on request from the corresponding author.
